# Clinical‐year veterinary students are most likely to be confident and competent in calving procedures after blending simulator practicals with videos

**DOI:** 10.1002/vetr.5774

**Published:** 2025-12-03

**Authors:** Jayne Orr, Robert F. Kelly, Monika Mihm Carmichael

**Affiliations:** ^1^ Scottish Centre for Production Animal Health and Food Safety, School of Biodiversity, One Health and Veterinary Medicine, University of Glasgow Glasgow UK; ^2^ Division of Veterinary Biomedical Sciences Royal (Dick) School of Veterinary Studies and the Roslin Institute Midlothian UK

**Keywords:** blended learning, calving, cow, simulator, student, veterinary

## Abstract

**Background:**

Veterinary students require safer practice of calving scenarios; however, the effects of a high‐fidelity calving simulator (SIM) practical and/or videos (computer‐assisted learning [CAL]) on student calving confidence (CONF) and competence (COMP) are unknown.

**Methods:**

Fourth‐year veterinary students received no teaching beyond previous lectures (LEC, *n* = 60), CAL (*n* = 59), SIM (*n* = 96) or CAL&SIM (*n* = 85). Students scored their CONF in 13 individual calving tasks before and after teaching delivery and were subsequently skill tested by internal (faculty) or external/peer/technical OSCE (Objective Structured Clinical Examination) assessors (non‐faculty).

**Results:**

Higher cumulative CONF scores required practical exposure (SIM 42.3 [95% confidence interval, CI 40.9–43.8], CAL&SIM 44.3 [95% CI 42.8–45.7] vs. LEC 33.3 [95% CI 31.2–35.5] and CAL 35.4 [95% CI 33.5–37.4]; *p* < 0.05), while videos already improved the OSCE pass rate (CAL 73%, SIM 84%, CAL&SIM 87% vs. LEC 40%; *p* < 0.05).

Blending CAL&SIM achieved the highest odds of students being ‘confident/very confident’ (odds ratio [OR] 18.5, *p* < 0.001) and passing the OSCE (OR 11.8, *p* < 0.001) with smaller positive effects of experience and baseline confidence on CONF or non‐faculty assessors on COMP (OR 2‒4, *p* < 0.01).

**Limitations:**

Benefits of improved student CONF and COMP in real‐life calving scenarios are likely but unknown.

**Conclusions:**

Best preparation for managing calving scenarios is blending simulator videos with the practical.

## INTRODUCTION

New graduates in farm animal practice are expected to attend an out‐of‐hour call to calve a cow with no immediate support, and pressure for a successful outcome. However, only 19% of graduates indicated that they could execute a delivery, and it took the new graduates 10 months to execute obstetrics without supervision.^1^ The Royal College of Veterinary Surgeons (RCVS), along with many other veterinary programme accrediting bodies, describes attending an animal in an emergency as a Day One (graduate) competence,^2^, ^3^, ^4^, ^5^ and farm animal practitioners in the UK also regard ‘assessing an obstetrical problem’ and ‘using a calving jack to deliver a calf’ as important skills.^6^ Clearly, there is a mismatch between what is expected from new graduates by practitioners, and what competences in bovine obstetrics graduates are equipped with. Thus, research into the optimum way to teach calving skills is needed.

While skill acquisition leading to competence is crucial, the appropriate level of confidence required by a student or new graduate to carry out a calving has not yet been established. Confidence is a judgement that influences whether an individual is willing (or not) to undertake an activity,^7^ but having too much or too little confidence can put patients in danger.^8^ The relationship between confidence and competence is unclear, either showing a correlation^9^, ^10^ or reporting it as poor,^11^, ^12^, ^13^, ^14^ but both need to be assimilated and considered together to educate capable veterinarians.^15^ Although one or the other may be predominant depending on the working environment, availability of back‐up, or complexity of the clinical scenario, ideally they mirror each other.^15^

Veterinary students traditionally learned how to calve cows from intramural lecture delivery and clinical case exposure during their extra mural studies (EMS). However, EMS is variable in quality, unpredictable, possibly high stakes and less accessible.^16^ In contrast clinical practical training using simulators provides a standardised learning opportunity that allows students safe and low‐stakes skills practice.^17^, ^18^ Veterinary simulators can have a positive impact on student confidence and competence in large animal^19^, ^20^, ^21^, ^22^, ^23^, ^24^ and small animal clinical skills,^25^, ^26^, ^27^, ^28^ due to the low‐stakes nature of practised scenarios. Skills and confidence required for the rectal examination of the reproductive tract in cows^19^ and mares,^21^ and for obstetrical manipulations in large animals^24^ were enhanced in students practising with haptic, computer‐controlled devices and relatively low‐fidelity simulator models. Although a high‐fidelity bovine obstetric simulator was used by experts to detail the steps, cognitive tasks and clinical decisions required for a successful calving, so the teaching of calving procedures to novices becomes more effective,^29^ the use of such a simulator in realistic but low‐stakes scenarios requires evaluation.

Blended learning combines ‘face‐to‐face interactions with online activities’,^30^ and increasingly, online, remote and asynchronous teaching modalities are blended with more traditional methods to enhance active learning.^31^ One study investigated the effect on students of using a veterinary simulator within a blended approach, thus integrating simulator video demonstrations into the practical‐sensory teaching of clinical examination skills.^32^ We are not aware of investigations using a high‐fidelity bovine obstetric simulator within a blended approach, where the success of video demonstrations of calving scenarios is evaluated against practical tuition, role playing, physical sensory and direct observational skill practice. Therefore, the main aim of this study was to investigate the impact of implementing a calving simulator model, on its own or as part of a blended learning approach, on the calving confidence and competence of clinical veterinary students in year 4 of a 5‐year programme. Because we know that student background affects baseline calving confidence of veterinary students,^33^ both likely influencing how students subsequently benefit from teaching, an additional aim was to determine effects of student characteristics on calving confidence and the outcome of a skills test (as a proxy for competence) after teaching delivery.

## MATERIALS AND METHODS

### Context

This study was conducted at the University of Glasgow (UOG), and the educational context was described in our previous study.^33^ Briefly, topics relevant to animal reproduction and obstetrics in the different species are delivered from years 1 to 4 during production animal and reproduction modules before students enter the mostly clinical and practical final year. For farm animal species, students learn the normal reproductive anatomy and physiology with normal parturition lectures and lambing practical classes in first year and then have three dedicated, 1‐hour lectures on calving cows in the third year. In the fourth year, all students attend a scheduled practical calving class using a high‐fidelity calving simulator in the production animal and clinical reproduction modules as described below. Students access information and resources, and carry out all their online activities via the virtual learning environment (VLE), Moodle 2, where access to teaching materials can be controlled using restrictions.

At the time of the study, the students had undertaken 12 weeks preclinical, and approximately 16 weeks clinical EMS. Undertaking EMS is compulsory and an RCVS requirement but is self‐directed and may not include calving a cow.^33^ However, all students will have undertaken a 2‐week preclinical lambing EMS placement.

### Study design overview

The study was conducted during the first 3.5 weeks (the ‘experimental period’) of a 6‐week farm animal teaching period that spanned two modules (4 weeks of ‘ruminant production’ and 2 weeks of ‘reproduction and fertility’) in three consecutive fourth‐year cohorts from 2016. There were 347 students eligible to take part (based on class list numbers) over the three study years.

In each study year, students were allocated to one of four different teaching groups, which defined access to further online and/or practical teaching during the experimental period. After being informed about the study in the module introduction session, students willing to participate provided written consent and completed the before teaching questionnaire (BTQ). Following teaching delivery at the end of the experimental period, students from all four teaching groups took part in a formative calving OSCE and completed the after teaching questionnaire (ATQ) during OSCE registration before the skills test. All students received access to all teaching materials and practicals after the end of the experimental period.

### Teaching groups

The four teaching groups are shown in Table 1.

**TABLE 1:** Teaching group name, description and number of fourth‐year clinical veterinary students allocated to each teaching group.

Teaching group name	Teaching group description	Study year (% of year allocated to each teaching group)			Total number of students allocated to each teaching group (% of total students)
		2016	2017	2018	
LEC	No further teaching over and above the specific cow calving lectures that were delivered in third year (8 months previously)	21 (18%)	22 (18%)	24 (22%)	67 (19%)
CAL	Access to calving video resources	27 (23%)	25 (21%)	21 (19%)	73 (21%)
SIM	Attendance at the practical class using the calving simulator	34 (29%)	38 (32%)	35 (32%)	107 (31%)
CAL&SIM	Access to the video resources and attendance at the practical class	34 (29%)	35 (29%)	31 (28%)	100 (29%)
Total number of students in each study year and in total		116	120	111	347

Abbreviations: CAL, computer‐assisted learning; LEC, lectures; SIM, simulator.

Calving procedures demonstrated in the simulator videos (CAL), and demonstrated and practised in the SIM, were based on the authors’ experience as farm animal practitioners and lecturers, and were guided by the individual steps and tasks identified by cognitive task analysis.^29^ The CAL resources consisted of five individual videos, 20 minutes total duration, demonstrating all procedural aspects of calving a cow (such as applying ropes, determining if there is sufficient room, extracting the calf and looking after the newborn calf; see also specific confidence questions in the questionnaire) using the high‐fidelity calving simulator and delivered by a farm animal lecturer. Access to the CAL was controlled via group permissions within the VLE.

**FIGURE 1:**
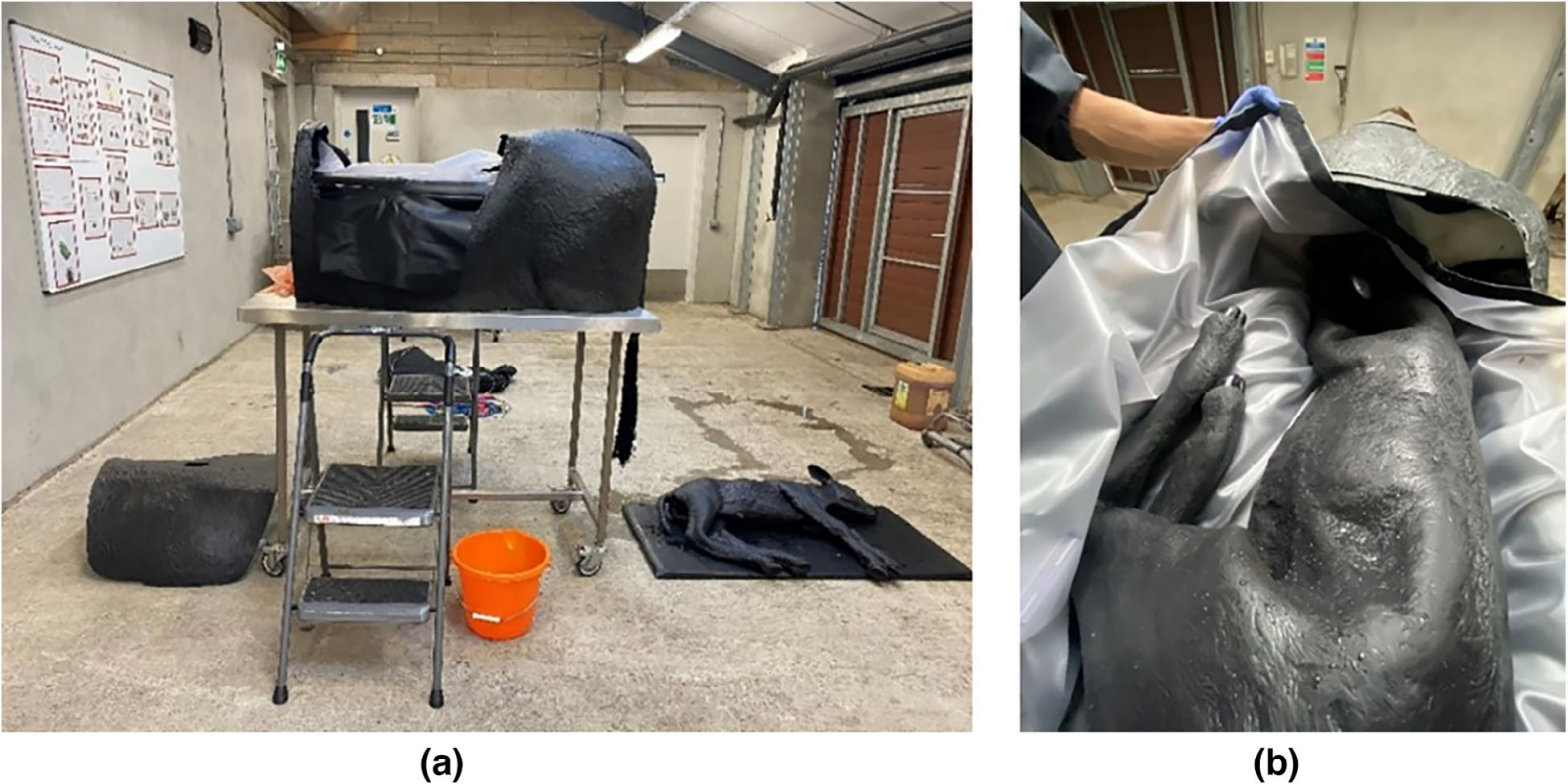
Calving simulator class set up: (a) cow body mounted on a table, calf, landing mat, bucket of obstetrical lubricant and ladders (to allow students to see inside and easier loading of the calf). (b) Simulator viewed from above to see calf in situ within the uterus.

For the SIM, students attended a 1‐hour 15‐minute practical class with one tutor (who followed a prescribed teaching plan), a maximum of six students and two identical calving simulators. The calving simulator cow and calf models were high‐fidelity and life sized and are commercially available from Veterinary Simulator Industries (www.vetsimulators.com/products#bovine, Figure 1). The cow model consists of a fibre glass cow body mounted on a table to mimic the height of a real cow. It contained an anatomically correct pelvis, inflatable air bags (to mimic abdominal and pelvic fat) and an accessible and visible vinyl bag acting as the uterus. The fully articulated, life‐sized calf weighing 22.7 kg was placed inside the cow simulator in various malpresentations, positions and postures, and can be manipulated and extracted using human force or with a calving aid by using ropes attached to the calf's legs and/or head. The calf could also be used to practise resuscitation. In addition to all procedural aspects of calving a cow, students also practised evaluating the cow's health status, history taking and communication with the farmer (using role play), and discussed immediate postpartum complications in the cow.

### Questionnaires

These were delivered to students on paper as previously described.^33^ Briefly, the BTQ established student characteristics, such as gender, year of birth, continent of origin, intention regarding area of veterinary practice they will pursue following graduation (mixed, small, equine, farm animal practice, etc.), prior calving experience, and a baseline calving confidence, which students answered on a Likert scale of 1‒5 (1—no confidence, 2—little, 3—some, 4—confident, 5—very confident) in 13 individual calving tasks required for a successful calving.^29^ The ATQ contained the same calving confidence questions as the BTQ. A copy of the BTQ is provided in the Supporting Information.

Students were asked to provide a personal identifier (last four digits of their matriculation number and first letter of their surname) when filling in BTQ and ATQ to match the demographic and confidence information from both questionnaires and to link this with the formative calving OSCE outcome.

### OSCE

A formative calving OSCE was designed to assess student competence in calving a cow with an anterior presentation, dorsosacral position, and one of two malpostures, head back (2016) and leg back (2017 and 2018) (see Supporting Information). All students from the cohort had a formative OSCE on the same day and there was no holding system in place. Each student had 1‐minute instruction reading time and 5‐minute OSCE task time. Based on a checklist of items the assessor awarded a preliminary score (out of 20) and global rating regardless of the total checklist score (fail, borderline [borderline fail and borderline pass used in 2018], pass and excellent).

Over the three years, OSCEs were assessed by four clinical academic veterinarians, one farm animal technician, one external veterinarian and four final‐year student peer assessors (supervised by a trained OSCE assessor). All had previously received half a day of training, had assessed multiple OSCEs before, were provided with a marking guide, and had opportunities to discuss assessments between OSCEs. Results were recorded on paper and both the numerical score and global rating (other than ‘excellent’) were moderated and agreed by J.O. and M.M.C. using a final marking guide (confidentially available on request), followed by the borderline regression method to calculate the pass mark for each cohort.^34^ Based on the pass mark, a final binary OSCE outcome of fail (below the pass mark) or pass (the pass mark or higher) was established for each student; excellent ratings remained unchanged.

## Data analysis

Data from the ATQ, BTQ and formative OSCE were entered into Microsoft Excel (version 2405 Build 16.0.17628.20006). ‘year of birth’ was converted into age by subtracting the year of birth from the study year.

### Categorisation of student background and confidence data

Student background variables (categorised as described previously),^33^ OSCE outcome and assessor categorisation are shown in Table 2. Briefly, a total experience score was calculated for each student based on the number of calvings they had (a) observed, (b) assisted with or (c) carried out unassisted, with an increasing score indicating more experience and independence.

**TABLE 2:** Categorisation of fourth‐year veterinary students based on their background variables obtained from questionnaire responses, OSCE outcome and which OSCE assessor they had.

Question/parameter	Responses	Categorisation
What is your intention following graduation?	Farm animal	Would encounter a cow
	Mixed	
	Equine	Would not encounter a cow
	Small animal	
	Exotics	
	Wildlife	
	Non‐clinical	
	Do not know	
Calving experience total score	= or less than 4 (only observed calvings)	None or minimal calving experience
	5–24 (assisted with at least 1 calving)^a^	Some calving experience
Total calving confidence score before teaching (BTQ)	14–26^b^	Little confidence
	27–65^c^	Some or more confidence
Total calving confidence score after teaching (ATQ)	14–39^d^	Little/some confidence
	40–65^e^	Confident/very confident
OSCE outcome	< pass mark	Fail
	= or > pass mark	Pass
OSCE assessors	UOG veterinarians	Faculty staff
	UOG farm animal technician, external veterinary tutors and final‐year students	Non‐faculty staff

Abbreviations: ATQ, after teaching questionnaire; BTQ, before teaching questionnaire; OSCE, Objective Structured Clinical Examination; UOG, University of Glasgow.

^a^ Students had assisted in at least 1‒2 calvings or even carried out calvings unassisted, and not just observed calvings.

^b^ Students self‐rated an average of Likert scale 1 or 2 (having ‘no’ or ‘little’ confidence) in all 13 calving tasks. No students gave Likert scale of 1 for all 13 tasks; hence, lowest score is 14.

^c^ Students self‐rated Likert scale 3 (having ‘some’ confidence), 4 (‘confident’) or 5 (‘very confident’) in at least one of the 13 calving tasks.

^d^ Students scoring none, little or some (Likert scale 1, 2 or 3) confidence on average in all calving tasks. No students gave Likert scale of 1 for all 13 tasks; hence, lowest score is 14.

^e^ Students scoring ‘confident’ or ‘very confident’ (Likert scale 4 or 5) on average in one or more of the calving tasks.

A total cumulative calving confidence score was then computed per student from the Likert ratings given to each of the 13 confidence questions,^33^ allowing numerical comparisons between BTQ and ATQ. An initial histogram of total confidence scores clearly indicated that the data could be divided into binary confidence categories, allowing logistic regression approaches. However, these differed between BTQ (students with ‘none’ or ‘little’ vs. ‘some or more’ confidence) and ATQ (students rating as ‘confident/very confident’ in at least one of the 13 tasks vs. those with only ‘little/some’ confidence) (Table 2).

### Statistical approaches

Descriptive data analysis and statistical analysis were carried out in Minitab 19.2020.1 and 21.4.1. Numerical data were checked for normality and either ANOVA or a paired *t*‐test (for normally distributed data), or Mann‒Whitney, Kruskal‒Wallis or Wilcoxon signed rank test (for not normally distributed data) were used. Categorical data were analysed using the chi‐square or Fisher's exact test (if expected counts in chi‐square test were <5). Significance levels were set as *p*‐value of less than 0.05 unless otherwise stated and a tendency was indicated at *p*‐value of less than 0.1.

Logistic regression was used to determine which teaching method was most likely to lead to a veterinary student (1) being confident/very confident in calving a cow (ATQ data), and (2) passing a calving competence skills test (OSCE data). In addition, any effects of study year or student background variables on being confident or competent after teaching delivery were quantified. Univariate logistic regression screened for plausible explanatory variables to be included in the multivariable model if their *p*‐value was less than 0.2.^35^ Possible confounding effects of excluded variables were also checked,^36^ and in three analyses, a 20%‒30% change in the equation coefficients for one of the final model variables was detected (see result description). Additionally, plausible interaction terms between variables that remained in the final model were checked, but no significant interaction terms were found for either of the two final models.

Data from respondents falling into demographic categories with very low numbers, specifically, would rather not say for gender (*n* = 1) and African students for continent of origin (*n* = 6), were excluded as they could not be grouped with any of the other categories. The variable ‘OSCE assessor’ was included in the OSCE analyses due to the known effect of assessors on the assessment outcome.^37^

## RESULTS

Of the 347 eligible students, 300 (86%) gave consent to take part in the BTQ (75% in 2016, 95% in 2017 and 89% in 2018), and of those 284 filled in the ATQ. Demographics of consenting students are provided in Table S1.

### Calving confidence

#### Effect of teaching method on total confidence scores and proportion of confident/very confident students

**FIGURE 2:**
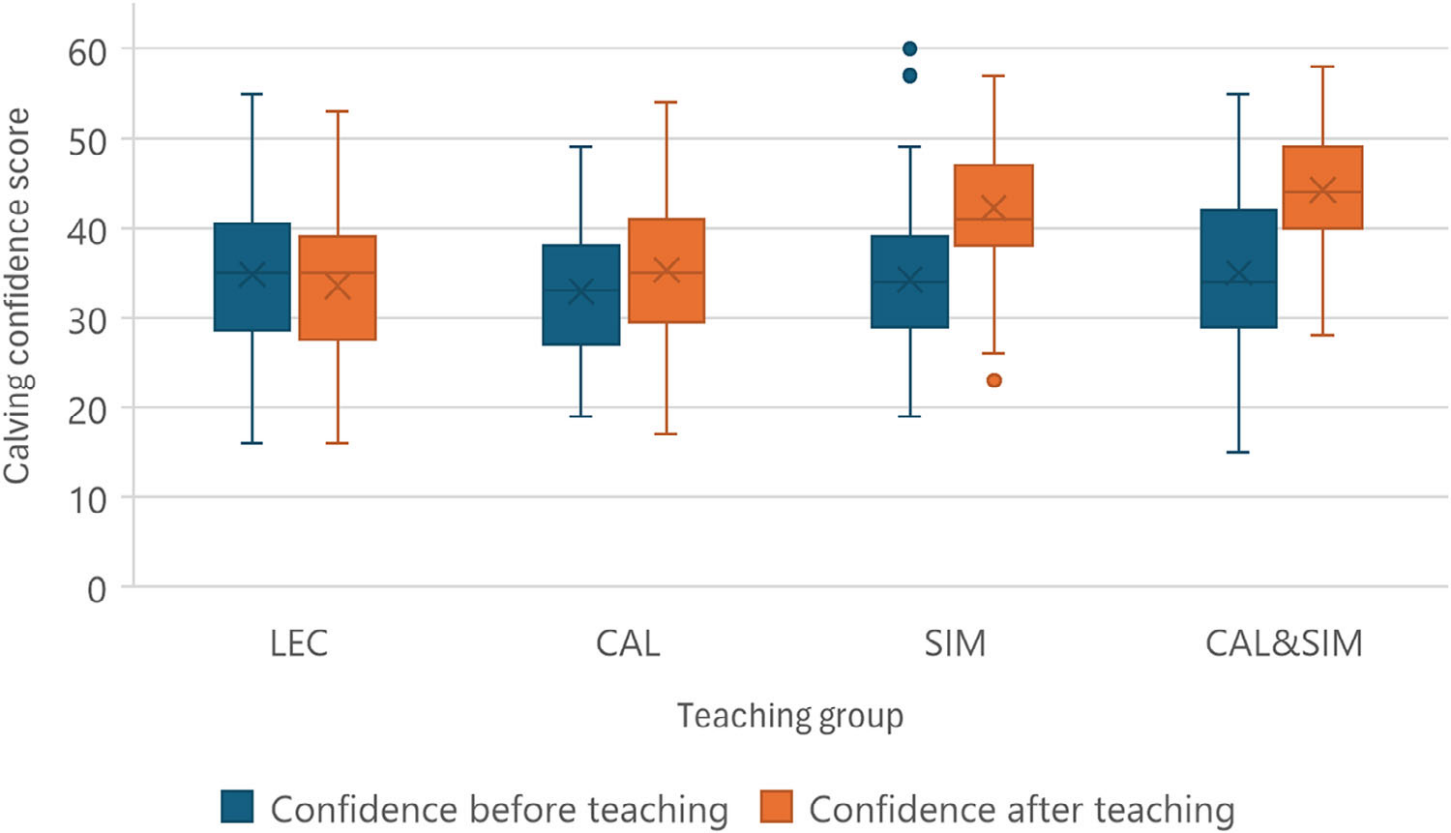
Boxplots displaying the median (line), mean (×), 75th percentile (box) and 95th percentile (error bars) for total cumulative confidence scores determined by summing the self‐rated confidence (on a Likert scale of 1‒5) in 13 individual skills required for calving cows before (blue) and after teaching (red) in fourth‐year clinical veterinary students.

The total cumulative calving confidence score for all consenting students was relatively low at the beginning of the experimental period (mean ± SD 34.3 ± 8.23, 95% confidence interval [CI] 33.41‒35.29), reflecting ‘little’ (=2 on the Likert scale of 1‒5) or ‘some’ (=3 on the Likert scale) confidence in the 13 calving tasks, but increased to 39.77 ± 8.39 (95% CI 38.79‒40.75) after teaching, reflecting on average ‘some’ (=3 on the Likert scale) confidence in all calving tasks (*p* < 0.01, maximum score = 65). Figure 2 shows the before and after teaching calving confidence scores for students in each teaching group. The largest increase in total confidence score after teaching was seen in students experiencing the blended approach CAL&SIM (*p* < 0.01) and also with SIM teaching alone (*p* < 0.05), while CAL students showed a tendency to have higher after teaching confidence (*p* < 0.1) and lecture (LEC) students had no change (*p* = 0.405). When comparing after teaching confidence scores between teaching groups, only the SIM practical led to higher scores (SIM and CAL&SIM vs. LEC, *p* < 0.05), with no effect of calving videos (CAL vs. LEC, *p* > 0.2; CAL&SIM vs. SIM, *p* > 0.1). The LEC group also had the highest proportion of students categorised as having little/some confidence (78%) while the CAL&SIM group had the highest proportion of confident/very confident students (78%).

#### Effects of teaching group and demographic variables on the likelihood of having calving confidence after teaching

Data from 277 ATQ responses were used for this part of the analysis. Table S2 shows the results of the univariate analyses for the outcome ‘confident/very confident’. Higher odds of being categorised as confident/very confident were achieved with the simulator demonstration videos or the SIM practical or both (compared with LEC, *p* < 0.2). The following background variables also individually increased (*p* < 0.2) the odds of being categorised as confident/very confident after teaching: being a declared male (compared with declared female); coming from Europe or North America (compared with Asia); intending to work with cows following graduation (compared with not); having some calving experience (compared with none/minimal); and having at least ‘some’ baseline confidence (compared with little).

Logistic regression results from the final model are shown in Table 3. The odds for veterinary students being categorised as confident/very confident in calving cows after teaching were two‐ to threefold higher when students were given access to the simulator videos (compared with LEC), or had some prior experience or confidence before teaching. The odds were elevenfold higher when students experienced the SIM practical, with a further large increase when students experienced the blended learning approach CAL&SIM. A small confounding effect of ‘intention to work with cows’ and ‘continent of origin’ on the positive effects of having ‘some experience’ on student confidence was identified. Model quality tests indicated good model fit (see Table S3).

**TABLE 3:** Final multiple logistic regression model to determine effects of teaching method, demographic variables and before teaching confidence on fourth‐year clinical veterinary students being confident/very confident in calving after teaching.

Variable	Odds ratio (95% confidence interval)	*p*‐Value
Teaching group		
LEC	Reference	–
CAL	2.10 (0.83–5.36)	0.119
SIM	10.89 (4.55–26.10)	0.000
CAL&SIM	18.45 (7.44–45.76)	0.000
Calving experience		
None/minimal	Reference	–
Something	2.49 (1.34–4.64)	0.004
Before teaching confidence (BTQ)		
Little	Reference	–
Some or more confidence	2.915 (1.34–6.32)	0.007

Abbreviations: BTQ, before teaching questionnaire; CAL, computer‐assisted learning; LEC, lecture; SIM, simulator.

### Calving competence (OSCE)

#### OSCE scores and pass rate

A total of 298 consenting students attended the formative OSCE. The mean ± SD OSCE numerical score for all 3 years and all teaching groups was 14.7 out of 20 (±4.34, 95% CI 14.23‒15.22), and was lower in LEC (11.3 ± 11.5, 95% CI 10.02‒12.55) compared with CAL (14.7 ± 4.25, 95% CI 13.57‒15.79), SIM (15.7 ± 3.73, 95% CI 14.92‒16.43) and CAL&SIM (16.1 ± 3.23, 95% CI 15.43‒16.83, *p* < 0.05).

**FIGURE 3:**
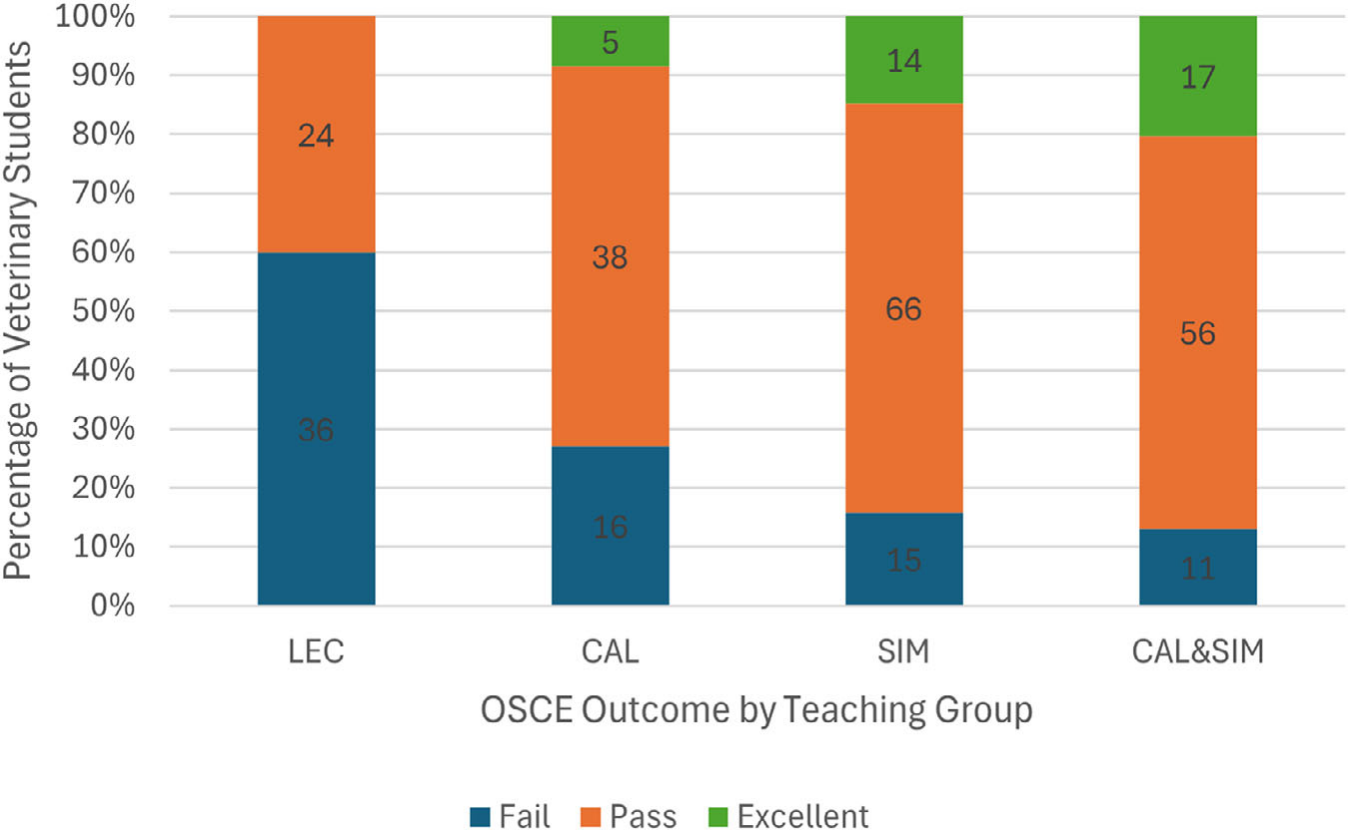
Proportion of fourth‐year clinical veterinary students with a calving skill OSCE outcome of ‘fail’, ’pass’ or ‘excellent’ at the end of the experimental period during which they received no teaching beyond third‐year lectures (LEC) or were exposed to calving simulator demonstration videos (computer‐assisted learning [CAL]), the calving simulator (SIM) practical or the blended approach (CAL&SIM) (data label is number of students per OSCE outcome). OSCE, Objective Structured Clinical Examination

Overall, 220 (74%) students passed the OSCE (36 of those who passed being awarded an excellent global rating) and 78 (26%) failed. The proportion of students who passed the OSCE was lower (*p* < 0.05) in the LEC teaching group compared to all other teaching groups (Figure 3). Adding the SIM practical to the CAL led to a higher proportion of passes (*p* = 0.035), but the proportion of passes was similar between the SIM and CAL&SIM groups (*p* = 0.61). Having the SIM practical on its own tended to lead to more students passing the calving OSCE compared with video resources alone (CAL, *p* = 0.088).

#### Relationship between after teaching calving confidence and OSCE outcome

Students who failed their OSCE skills test had a lower total confidence score (36.08 ± 8.54, 95% CI 34.09‒38.08) than students who passed the OSCE (41.04 ± 7.97, 95% CI 39.96–42.13, *p* < 0.05). Similarly, when students had not received the SIM practical (LEC and CAL students) but were ‘confident/very confident’ in calving cows they tended to achieve a higher OSCE pass rate (21/29 vs. 44/83 students with ‘little/some’ confidence, *p* = 0.068).

#### Effects of teaching group and student variables on OSCE outcome

Data from 291 students were used for this part of the analysis. Table S4 shows the results of the univariate analyses for the outcome ‘OSCE Pass’. Being exposed to the simulator demonstration videos, the practical, or both increased (*p* < 0.2) the odds of passing the calving OSCE (compared with LEC). The following background variables also individually increased (*p* < 0.2) the odds of passing the calving OSCE and were thus included in the multivariable logistic regression model: being a declared male (compared with declared female); coming from Europe (compared with Asia); being confident/very confident after teaching (compared with no/little/some confidence); being a non‐faculty assessor (compared with a faculty veterinarian).

Logistic regression results from the final model are shown in Table 4. Model details including model fit are presented in Table S3. Access to the calving simulator videos alone (CAL) increased the odds for students passing the calving OSCE compared to no further teaching (LEC). However, the learning experience of the SIM practical almost doubled such odds, and the blended learning approach with CAL exposure prior to the SIM practical led to the highest odds of passing the calving OSCE. A small confounding effect of ‘gender’ on the enhancing effect of the ‘CAL teaching group’ on passing the OSCE was identified.

**TABLE 4:** Final multiple logistic regression model to determine effects of calving teaching method and OSCE assessor on the OSCE outcome ‘pass’ at the end of the experimental period during which fourth‐year clinical veterinary students received either no further teaching beyond third‐yearlectures LEC), had access to video demonstrations (computer‐assisted learning [CAL]), were scheduled for a practical calving class (simulator [SIM]) or had the blended approach (CAL&SIM).

Variable		Odds ratio (95% confidence interval)	*p*‐Value
Teaching group	LEC	Reference	‒
	CAL	5.11 (2.12–12.32)	<0.001
	SIM	8.84 (3.84–20.36)	<0.001
	CAL&SIM	11.79 (4.85–28.66)	<0.001
OSCE assessor	Faculty	Reference	‒
	Non‐faculty	4.22 (2.04–8.73)	<0.001

## DISCUSSION

This study showed that implementation of a calving simulator in clinical veterinary education had a positive impact on student calving confidence and competence. Using the simulator to make video demonstration resources for students already enhanced their OSCE pass rate, but physical access to the simulator was crucial to increase their total calving confidence scores. Blending video resources with the practical, however, achieved the highest odds of students being categorised as confident/very confident in calving cows.

There are numerous studies reporting positive competence outcomes (with confidence studies being less common) following student interaction with simulators for veterinary clinical skills^20^, ^24^, ^27^, ^38^ and for medical obstetrics skills.^39^, ^40^, ^41^, ^42^ A much smaller scale study to ours showed that confidence and knowledge improved in large animal obstetrics after access to a low‐fidelity simulator.^24^ Unfortunately, the consequences of such simulator teaching on student skills were not reported.

We provide clear evidence for positive effects of veterinary simulation in a blended approach on student calving competence, similar to an equally sized study of first‐year veterinary students who were taught cat clinical examination using the blended approach and showed improved competence scores for some clinical examination elements.^32^ However, there were also negative effects seen likely impacting student confidence (being more afraid and being concerned about being wounded). In contrast, we provide evidence for positive confidence effects following the blended teaching delivery, where confidence ratings increased in all individual calving tasks. The negative confidence effects in aspects of cat examination may be explained by their inclusion of live examinations, which are higher stakes.

Similar to our study showing a positive impact of the online video demonstrations, particularly on calving skill test outcome, improved examination results and self‐efficacy following exposure to three‐dimensional resources for equine parturition have also been reported^38^, ^43^ (albeit not directly comparable to the bovine obstetrics OSCE). Contradictory results were observed in undergraduate student nurses given access to OSCE video exemplars leading to increased engagement, and reduced anxiety, but, surprisingly, no change in OSCE performance.^44^ While in the two cited studies, higher confidence following exposure to demonstration material was seen, but not necessarily improved clinical competence, our study revealed the diverse interplay between student confidence and competence: we saw a more direct positive effect of video resources on the calving skills than on calving confidence scores, but we also saw a positive effect of higher confidence on the OSCE pass rate in students who had not received the practical. Clearly, this interaction requires further investigations in veterinary students, similar to medical students.^8^

We have previously reported the influence of veterinary student background on baseline calving confidence,^33^ and our study revealed maintained background effects on confidence after teaching, but there are very few reports of the influence of demographic factors on clinical competence. Here, calving simulator training determined OSCE pass rate, not calving confidence, previous experience or intention to work with cows after graduation. Despite random allocation to the different teaching groups, the CAL teaching group had more males, which may explain why gender had a small confounding effect on the CAL OSCE outcome. In contrast to our study, prior experience (with a veterinarian) increased student ability to identify pregnant cows using transrectal palpation.^45^ Similarly, student background (if they were from the city, farm or mixed) also affected such diagnostic competence, and this still requires to be investigated in relation to calving competence.

Prior experience and baseline confidence had a smaller effect than teaching method but still increased the odds of students being categorised as (very) confident. There is likely a close relationship between the opportunities students have in relation to farm animal work, subsequent calving experience, and developing the intention to work with cows, which may account for the small confounding effects identified. Understanding why individuals go into farm animal practice and what can overcome the challenges faced by individuals from a more urban background is worth exploring further to ensure adequate veterinary care in rural communities. Farm animal EMS will thus play an important role by allowing students to gain confidence through prior experience with live calvings. Conversely, the recent COVID‐19 pandemic influencing both student mental health and EMS experience may have reduced calving confidence of recent graduates.

We did not anticipate the OSCE assessor effect due to measures taken to ensure consistency, but this effect has been reported previously.^37^ Further analysis of OSCE checklist scores showed that the score from non‐faculty assessors was more than three standard errors above the overall mean score,^46^ indicating we had strict ‘hawks’ and lenient ‘doves’.^47^ In future studies, the number of students in each teaching group assessed by each assessor category should be similar and OSCE assessment training may need to further focus on consistency.

Despite timetabling challenges, the design allowed dissecting the effects of video simulator demonstrations from those gained by practical exposure, with all students subsequently receiving all teaching. The BTQ and ATQ were delivered on paper in class, resulting in a high response rate (over 80%), and attendance at the formative OSCE was also very high (298/300 consenting students). The study included three different fourth‐year student cohorts to determine consistent effects of the different teaching methods, and both OSCE and confidence questions were designed around a published list of calving skills.^29^ Limitations were the binary categorisation of data for some of the analyses, which could have diluted some of the granularity of the data, and lack of validation of the calving simulator with external farm animal practitioners. Repetition improves acquisition of complex veterinary skills,^48^ and repeated simulation benefits student confidence.^49^ In our study CAL&SIM students had two formal opportunities to engage with calving learning materials, compared with only one intervention in the CAL or SIM students. Currently, it is not known whether the repetition or the unique combination of video material with SIM practical exposure led to highest confidence and skill test outcomes in our study, but we speculate that repeated SIM or live calving exposure would have most benefits.^49^ Furthermore, it is not known how student confidence and competence evaluated in an OSCE during this study compares to the capability of a student faced with a real calving in a clinical setting, where animal‐based outcomes still require to be measured.^50^

This study adds further evidence that simulation benefits clinical skills but, importantly, also student confidence, and should help inform faculty decisions regarding implementation of similar simulators to aid the training of their veterinary students in the high stake, composite skill of calving cows. Our findings also support use of digital simulator resources. It is hoped the graduates in this study will have confidence in their calving skills and feel they are more prepared to deal with a calving emergency.

Our study and others support a correlation between having confidence and passing a skills test,^9^, ^10^ while others do not,^11^, ^14^, ^48^, ^49^ but it is re‐assuring for our less‐confident students that they can acquire calving skills if they have received simulator training. However, the thresholds of confidence and competence to ensure capable graduates are unknown.^15^ In the future, we require to assess impact and duration of improved student calving confidence and competence in the field under a variety of obstetrical presentations and farm working environments, and possibly even show benefits to graduate retention in the sector. Changing specific elements of the SIM practical (e.g. use of actors) may also enhance outcomes by ensuring complete immersion in calving scenarios.

## AUTHOR CONTRIBUTIONS

Jayne Orr was responsible for conception, design, acquisition of data, analysis, interpretation, drafting of the manuscript and final approval. Robert F. Kelly was responsible for conception, design, interpretation, critical revision of the manuscript and final approval. Monika Mihm Carmichael was responsible for conception, design, acquisition of data, analysis, interpretation, critical revision of the manuscript and final approval.

## CONFLICT OF INTEREST STATEMENT

The authors declare they have no conflicts of interest.

## ETHICS STATEMENT

Ethical approval was granted from the UOG Ethics Committee, which also stipulates adherence to UK data protection legislation regulating secure storage and access to research data (application number 20016009). The students were informed about the study and advised participation was completely voluntary both via a forum post in the VLE and during a face‐to‐face module introduction session.

## Supporting information



Supporting Information

Supporting Information

Supporting Information

Supporting Information

Supporting Information

Supporting Information

## Data Availability

Data available on request from the authors.
