# Supplementary material for: Clinical‐year veterinary students are most likely to be confident and competent in calving procedures after blending simulator practicals with videos
Source: Vet Rec. 2025 Dec 3;198(1):e11–20. doi: 10.1002/vetr.5774 (PMC12758265; doi:10.1002/vetr.5774)
Supplement: Supplementary file 1 — Supporting Information [file VETR-198--s006.docx]

| **Demographic** | | **Number of students (percent) or descriptive statistics** |
| --- | --- | --- |
| Gender | Male | 67 (23%) |
|  | Female | 232 (77%) |
|  | WRNS* | 1 (0.3%) |
| Age | Mean  95% CI  +/- SD | 23.4  23.0 to 23.8  +/- 3.39 |
| Continent of origin | Europe | 162 (54%) |
|  | North America | 96 (32%) |
|  | Asia | 36 (12%) |
|  | Africa | 6 (2%) |
| Intention following graduation** | Would encounter cows | 127 (42%) |
|  | Would not encounter cows | 173 (58%) |
| Calving experience total score  (out of a maximum of 24)*** | Mean  95% CI  +/- SD | 6.7  6.00 to 7.37  +/- 6.03 |

Supplementary Table 1 – Demographics of 300 4^th^ year clinical veterinary students consenting to take part in the study. There was no difference in any of the demographic factors between the three study years (p > 0.05). Confidence interval (CI), standard deviation (SD)

* Would rather not say (WRNS): this option was given in 2018/19 only.

**see also Table 2 categorisation of data. Students were categorised depending on their responses to question 5 in the BTQ, where students choosing farm animal practice or mixed practice after graduation would encounter cows and, therefore, calving scenarios.

***see also Table 2 categorisation of data and description in our previous study (32). Students’ calving experience was converted into a numerical score depending on how many calvings they had 1. observed (scores 1-4), 2. assisted with (scores 5 to 8), or 3. carried out unassisted (scores 9-12), to a possible maximum of 24.
