# Supplementary material for: Clinical‐year veterinary students are most likely to be confident and competent in calving procedures after blending simulator practicals with videos
Source: Vet Rec. 2025 Dec 3;198(1):e11–20. doi: 10.1002/vetr.5774 (PMC12758265; doi:10.1002/vetr.5774)
Supplement: Supplementary file 2 — Supporting Information [file VETR-198--s005.docx]

Supplementary Table 2 Results from the univariate logistic regression analysis of possible explanatory demographic and teaching group variables for 4^th^ year clinical veterinary students being categorised as confident/very confident in calving cows at the end of the experimental period*. Number (and percentage) of students in the two confidence categories are presented (except for age in years). Confidence interval (CI), Standard deviation (SD)

| Variable | | calving confidence category | | | Odds Ratio  (95% Confidence interval | P value |
| --- | --- | --- | --- | --- | --- | --- |
|  |  | Little/  some  confidence | Confident/very  confident | Total |  |  |
|  |  | 126  (45%) | 151  (55%) | 277 |  |  |
| Teaching Group* | LEC | 42  (81%) | 10  (19%) | 52 | Reference | - |
|  | CAL | 38  (69%) | 17  (31%) | 55 | 1.88  (0.77 – 4.60) | 0.168 |
|  | SIM | 28  (32%) | 59  (68%) | 87 | 8.85  (3.89 – 20.16) | 0.000 |
|  | CAL&SIM | 18  (22%) | 65  (78%) | 83 | 15.17  (6.39 – 36.02) | 0.000 |
| Study year | 2016/17 | 42  (52%) | 39  (48%) | 81 | Reference | - |
|  | 2017/18 | 47  (44%) | 60  (56%) | 107 | 1.38  (0.77 - 2.45) | 0.282 |
|  | 2018/19 | 37  (42%) | 52  (58%) | 89 | 1.51  (0.83 – 2.78) | 0.180 |
| Age (years) | Mean  95% CI  +/- SD | 23.3  22.78 – 23.77  +/- 2.80 | 23.4  22.81 - 24.06  +/- 3.88 | NA | 1.01  (0.95 - 1.09) | 0.700 |
| Gender ^a^ | Female | 102  (48%) | 112  (52%) | 214 | Reference | - |
|  | Male | 24  (38%) | 39  (62%) | 63 | 1.48  (0.83– 2.63) | 0.181 |
| Continent | Asia | 24  (67%) | 12  (33%) | 36 | Reference | - |
|  | Europe | 58  (38%) | 93  (62%) | 151 | 3.21  (1.49 – 6.90) | 0.003 |
|  | North America | 44  (49%) | 46  (51%) | 90 | 2.09  (0.93 – 4.69) | 0.073 |
| Intention following graduation category | Would not encounter cows | 81  (51%) | 79  (49%) | 160 | Reference | - |
|  | Would encounter cows | 47  (40%) | 70  (60%) | 117 | 1.45  (0.90 – 2.35) | 0.129 |
| Calving experience category** ^b^ | None/  minimal | 55  (61%) | 35  (39%) | 90 | Reference | - |
|  | Some | 71  (38%) | 115  (62%) | 186 | 2.55  (1.52 – 4.27) | 0.000 |
| BTQ confidence category *** ^c^ | Little | 32  (67%) | 16 (33%) | 48 | Reference | - |
|  | Some or  more  confidence | 93  (41%) | 132  (59%) | 225 | 2.84  (1.47 – 5.47) | 0.002 |

^a^ 1 x would rather not say (WRNS), ^b^ 1 x no answer, ^c^ 4 x no answer

*See also methods description. During this period students received either 1. no teaching (LEC), 2. Online access to video demonstrations (CAL), 3. practical calving simulator training (SIM), or 4. the blended approach (video demonstrations and practical simulator training, CAL&SIM).

** See also methods description. Students were categorised depending on their cumulative numerical score given to the number of calvings 1. observed, 2. assisted with, and 3. carried out unassisted. Students with some experience had at least assisted in 1-2 calvings.

*** See also methods description. When students gave consent and filled in the survey at the beginning of the experimental period (Before Teaching Questionnaire, BTQ), they self-rated their confidence in 13 individual calving tasks (from Likert scale 1=none to 5=very confident). These BTQ ratings were summed to allow categorisation into students with none or little (Likert scale 1 or 2 on average in all tasks) versus students with some confidence (Likert scale 3 or more in at least one of the calving tasks).
