# Supplementary material for: Clinical‐year veterinary students are most likely to be confident and competent in calving procedures after blending simulator practicals with videos
Source: Vet Rec. 2025 Dec 3;198(1):e11–20. doi: 10.1002/vetr.5774 (PMC12758265; doi:10.1002/vetr.5774)
Supplement: Supplementary file 3 — Supporting Information [file VETR-198--s003.docx]

Supplementary table 3 Final logistic regression model parameters for the binary outcomes 4^th^ year clinical veterinary students 1. being confident in calving cows, and 2. passing the calving skills test, the formative OSCE. See methods description and Table 2 regarding the categorical variables included in the analyses.

|  | Confidence at the end of the experimental period (ATQ) | OSCE Outcome |
| --- | --- | --- |
| Final model equation | Confidence (confident/very confident versus no/Little/Some confidence) ~ Teaching group LEC + Teaching group CAL + Teaching group SIM + Teaching group CAL&SIM + Baseline confidence + Experience. | OSCE outcome (Pass versus Fail) ~ Teaching group LEC + Teaching group CAL + Teaching group SIM + Teaching group CAL&SIM + OSCE assessor |
| Akaike Information Criterion corrected (AICc) | 301.03 | 267.02 |
| Area under the ROC curve | 0.808 | 0.781 |
| Hosmer Lemeshow P value | 0.553 | 0.303 |
