# Supplementary material for: Clinical‐year veterinary students are most likely to be confident and competent in calving procedures after blending simulator practicals with videos
Source: Vet Rec. 2025 Dec 3;198(1):e11–20. doi: 10.1002/vetr.5774 (PMC12758265; doi:10.1002/vetr.5774)
Supplement: Supplementary file 4 — Supporting Information [file VETR-198--s004.docx]

Supplementary Table 4 Results from the univariate logistic regression analysis of possible explanatory demographic, confidence and teaching group variables for 4^th^ year clinical veterinary students passing the calving OSCE at the end of the experimental period*. Number (and percentage) of students in the OSCE outcome categories are presented (except for age in years). Confidence interval (CI), Standard deviation (SD)

| Variable | | OSCE outcome category | | | Odds Ratio  (95% confidence interval) | P value |
| --- | --- | --- | --- | --- | --- | --- |
|  |  | Fail  76  (26%) | Pass  215  (74%) | Total  291 |  |  |
| Teaching group* | LEC | 36  (62%) | 22  (38%) | 58 | Reference | - |
|  | CAL | 14  (25%) | 42  (75%) | 56 | 4.91  (2.20 – 10.97) | < 0.001 |
|  | SIM | 15  (16%) | 78  (84%) | 93 | 8.501  (3.96 – 18.30) | < 0.001 |
|  | CAL&SIM | 11  (13%) | 73  (87%) | 84 | 10.86  (4.75 – 24.82) | < 0.001 |
| Study Year | 2016/17 | 19 (23%) | 65  (83%) | 84 | Reference | - |
|  | 2017/18 | 31 (28%) | 80  (72%) | 111 | 0.75  (0.39 -1.46) | 0.401 |
|  | 2018/19 | 26 (27%) | 70  (73%) | 96 | 0.79  (0.40 - 1.56) | 0.491 |
| Age  (in years) | Mean  (95% CI,  +/- SD) | 23.5  (95% CI 22.7 – 24.2, +/- 3.2) | 23.3  (95% CI 22.9 – 23.8, +/- 3.5) | 23.4  (95% CI 23.0 – 23.8, +/- 3.4) | 0.99  (0.92 - 1.06) | 0.736 |
| Gender | Female | 65  (29%) | 163 (71%) | 228 | Reference | - |
|  | Male | 11  (17%) | 52  (83%) | 63 | 1.89  (0.93 – 3.84) | 0.081 |
| Continent | Asia | 13  (36%) | 23  (64%) | 36 | Reference | - |
|  | Europe | 39  (24%) | 121 (76%) | 160 | 1.75  (0.81 – 3.79) | 0.153 |
|  | North America | 24  (25%) | 71  (75%) | 95 | 1.67  (0.74 – 3.81) | 0.221 |
| Intention following graduation category | Would not encounter cows | 48  (29%) | 119 (71%) | 167 | Reference | - |
|  | Would encounter cows | 28 (23%) | 96  (77%) | 124 | 1.38  (0.81 – 2.37) | 0.238 |
| Experience category** ^a^ | None/  minimal | 27  (29%) | 65  (71%) | 92 | Reference | - |
|  | Something | 49  (25%) | 149  (75%) | 198 | 1.26  (0.73 – 2.20) | 0.408 |
| Before teaching confidence (BTQ)^b&^ | Little | 15  (30%) | 35  (70%) | 50 | Reference | - |
|  | Some/  Confident/very confident | 60  (25%) | 177  (75%) | 237 | 1.32  (0.65 – 2.48) | 0.494 |
| After teaching confidence (ATQ)^c&&^ | Little/  Some | 44  (35%) | 82  (65%) | 126 | Reference | - |
|  | Confident/very confident | 27  (18%) | 124 (82%) | 151 | 2.46  (1.42 – 4.29) | 0.001 |
| OSCE assessor | Faculty vets | 63  (34%) | 121 (66%) | 184 | Reference | - |
|  | Non-faculty | 13  (12%) | 94  (88%) | 107 | 3.77  (1.96 – 7.25) | < 0.001 |

^a^ 1 student missing data, ^b^ 4 students missing data, ^c^ 14 students missing data

*See also methods description. During the experimental period students received either 1. no teaching (LEC), 2. Online access to video demonstrations (CAL), 3. practical calving simulator training (SIM), or 4. the blended approach (video demonstrations and practical simulator training, CAL&SIM).

** See also methods description. Students were categorised depending on their cumulative numerical score given to the number of calvings 1. observed, 2. assisted with, and 3. carried out unassisted. Students with some experience had at least assisted in 1-2 calvings.

^&^ See also methods description. When students gave consent and filled in the survey at the beginning of the experimental period (Before Teaching Questionnaire, BTQ), they self-rated their confidence in 13 individual calving tasks from Likert scale 1=none to 5=very confident. These baseline confidence ratings were summed to allow categorisation into students with none or little confidence (Likert scale 1 or 2) on average in all tasks versus students with some (Likert scale 3) confidence in at least one of the calving tasks.

^&&^ See also methods description. When students filled in the confidence survey at the end of the experimental period just before the calving skills test (After Teaching Questionnaire, ATQ), they again self-rated their confidence in 13 individual calving tasks from Likert scale 1=none to 5=very confident. These confidence ratings were summed to allow categorisation into students with none, little or some confidence (Likert scale 1, 2 or 3) on average in all tasks versus students self-rating as confident or very confident (Likert scale 4 and 5) on average in one or more of the calving tasks.
