# Supplementary material for: Clinical‐year veterinary students are most likely to be confident and competent in calving procedures after blending simulator practicals with videos
Source: Vet Rec. 2025 Dec 3;198(1):e11–20. doi: 10.1002/vetr.5774 (PMC12758265; doi:10.1002/vetr.5774)
Supplement: Supplementary file 5 — Supporting Information [file VETR-198--s002.docx]

**Evaluation of calving simulator training in the veterinary undergraduate curriculum as part of a blended learning programme**

**Consent**

I give consent for my anonymized data to be used and understand that a unique identification number will only have to be used to match before and after questionnaires.

I understand that my skills may be assessed in a formative OSCE (BVMS4) or by the practice vet (BVMS5).

I understand I can withdraw at any time.

I have read the information sheet/attended information lecture and have taken the opportunity to ask any questions if necessary.

Tick box

## Questionnaire

### Background Information

1. ID number_____________________________

**(Last 4 digits of Matriculation number and first initial of surname)**

1. Gender (please circle)

Male Female Other Would rather not say

1. Year of birth________________________________
2. Continent of origin (please circle)

Asia Australasia Africa Europe North America South America

1. What is your intention following graduation? (please circle)

Small Equine Farm Mixed Non-clinical Don’t know

1. **Confidence level self-assessment**
2. How confident do you feel with the following tasks (a – m) when calving a cow? (Please tick one box for each of the listed tasks)

|  | **Confidence level** | | | | |
| --- | --- | --- | --- | --- | --- |
| **Tasks** | **No confidence** | **Little Confidence** | **Some confidence** | **Confident** | **Very confident** |
|  | **1**  **(not confident)** | **2** | **3** | **4** | **5**  **(very confident)** |
| 1. Restraint of the cow |  |  |  |  |  |
| 1. Evaluating the cow’s current health status |  |  |  |  |  |
| 1. History taking |  |  |  |  |  |
| 1. Preparing the cow for vaginal examination |  |  |  |  |  |
| 1. Palpation of vagina/cervix/fetus |  |  |  |  |  |
| 1. Coming to a conclusion about the obstetrical problem |  |  |  |  |  |
| 1. Correcting the obstetrical problem |  |  |  |  |  |
| 1. Determining if sufficient room to extract the calf |  |  |  |  |  |
| 1. Applying the head rope, leg ropes and calving aid |  |  |  |  |  |
| 1. Extracting the calf |  |  |  |  |  |
| 1. Reviving the calf |  |  |  |  |  |
| 1. Dealing with immediate postpartum complications in the cow (eg. bleeding) |  |  |  |  |  |
| 1. Communicating with the farmer |  |  |  |  |  |

### Previous Experience:

1. How many calvings needing assistance have you **observed** (Didn’t get to do anything practical)? (please circle)

0 1-2 3-5 6-10 10+

1. How many calvings needing assistance have you **helped** with (with some direction from vet/teacher/farmer)? (please circle)

0 1-2 3-5 6-10 10+

1. How many calvings needing assistance have you **carried out with no help** (i.e. no direction from vet/teacher/farmer)? (please circle)

0 1-2 3-5 6-10 10+
