# Supplementary material for: Clinical‐year veterinary students are most likely to be confident and competent in calving procedures after blending simulator practicals with videos
Source: Vet Rec. 2025 Dec 3;198(1):e11–20. doi: 10.1002/vetr.5774 (PMC12758265; doi:10.1002/vetr.5774)
Supplement: Supplementary file 6 — Supporting Information [file VETR-198--s001.docx]

| **Instructions to Assessor** | **Practical Skill Station**  **Calving Simulator** |
| --- | --- |
| - Indicate that this is a practical skills station - Ask the candidate to read the scenario and instructions - If the candidates begins the task without putting on gloves, direct them to do so (but they lose the mark) - Place the calf in the abnormal presentation between students – left leg bent cranial at carpus. Ensure the calf is engaged within the pelvis so the student needs to push it back to correct the leg.       N.B. Marks are allocated for the correct examination technique, correct findings and corrective measures, not for what the candidate states they are examining/doing. | |
| **Equipment Needed:**   - Disposable gloves (rectal) - Lubricant - Watered down lubricant in a 5L bucket (50:50) rubbed within the uterus and on calf (Top up as necessary). - Calving ropes (2x leg ropes, 1x head rope, 1x head wire) - Calving jack (same type each station) - Calving simulator on table with cushions filled firmly with air - Calf latex model or dead calf. - Paper towels - Clinical waste bin - Step ladders | |
| Fail = Leg remains in position OR Student very rough (would perforate the uterus) OR head rope strangles calf (TBC)  Borderline = Leg corrected but no ropes on legs OR student quite rough (risk of perforation of uterus)  Pass = Leg corrected and leg ropes applied | |
| **Instructions to Candidate** | **Practical Skill Station**  **Calving Simulator** |
| **This is a practical skill station**   - The calving simulator on the table represents a second parity cow that has an abnormal calving. An assistant is available if required.   **First:**   - Prepare for a vaginal examination in this cow (assume that you have washed your hands and the perineal region)   **Then:**   - **Palpate** the calf in the calving simulator - **Describe** the calf’s presentation - **Correct** any malpresentation - **Demonstrate** how you would attach the calf to the calving aid - **State** when you are ‘ready to start delivery of the calf’ - **Describe** how you would extract the calf using the calving aid   ****NOTE: You are not required to deliver the calf via the vagina**** | |

|  | **Assessor** | **Candidate** | | | **Station number**  XX | | |
| --- | --- | --- | --- | --- | --- | --- | --- |
| **Action/Response** | | | | | **Done/Correct** | | **Mark** |
| The Student:  Uses lubricant +/- gloves | | | | | **1** | |  |
| Palpates the calf’s head and both forelegs | | | | | **1** | |  |
| Correctly describes:   - Anterior position with left leg back | | | | | **1** | |  |
| Correctly re-positions the calf’s leg by:   - Pushing the calf back (give marks if don’t need to do this but moving leg is done safely) - Re-positions leg – brings leg/foot towards vulva (1 mark) and cups hoof with hand (2 marks) | | | | | **1**  **1/2/3** | |  |
| Places calving ropes TIGHTLY above the LEFT metacarpi (1 mark) and extends rope outwards of the vagina coming from ventral aspect of leg (1 mark) | | | | | **1/2** | |  |
| Places calving ropes TIGHTLY above the RIGHT metacarpi (1 mark) and extends rope outwards of the vagina coming from ventral aspect of leg (1 mark) | | | | | **1/2** | |  |
| Successfully applies a head rope in a safe position | | | | | **2** | |  |
| *Use of calving jack*   - Calving jack placed on rump beneath vulva - Attaches RIGHT leg - Attaches LEFT leg   (no extra marks for head)   - Describes correctly how to use the calving jack   (2 people, downward manner, work with cow safety) | | | | | **1**  **1**  **1**  **1** | |  |
| States after correctly repositioning and attaching ropes that ‘the calf is safe to deliver’ | | | | | **1** | |  |
| Communicates effectively with assistant | | | | | **1** | |  |
| Carries out procedures in an efficient and safe manner | | | | | **1** | |  |
| **Total** | | | | | **20** | |  |
| ***Candidate ran out of time (please record if this is the case)*** | | | | | | |  |
| ***Other comments:*** | | | | | | | |
| ***Global Rating:***  ***Fail*** | | | ***Borderline*** | ***Pass*** | | ***Excellent*** | |
